# Case Report: Pulmonary benign metastasizing leiomyoma with mild ^18^F-FDG uptake occurring 30 years after hysterectomy

**DOI:** 10.3389/fonc.2026.1934720

**Published:** 2026-09-09

**Authors:** Huifeng Zou, Shengjie Chen

**Affiliations:** 1 Department of Thoracic Surgery, The Affiliated Hospital of Jiangsu University, Zhenjiang, Jiangsu, China; 2 School of Medicine, Jiangsu University, Zhenjiang, Jiangsu, China

**Keywords:** 18F-FDG PET/CT, benign metastasizing leiomyoma, estrogen receptor, latency, pulmonary nodules, uterine leiomyoma

## Abstract

**Background:**

Benign metastasizing leiomyoma (BML) is a rare smooth-muscle tumor that occurs in patients with a history of uterine leiomyoma, with the lungs being the most commonly involved extrauterine site. Pulmonary BML can mimic metastatic malignancy on imaging. Decades-long intervals after uterine surgery have been reported, and ^18^F-FDG uptake may vary among lesions.

**Case presentation:**

A 59-year-old postmenopausal, nonsmoking woman presented with multiple bilateral pulmonary nodules 30 years after hysterectomy for uterine leiomyoma. ^18^F-FDG PET/CT demonstrated mild FDG uptake in the dominant pulmonary lesion, with a maximum standardized uptake value (SUVmax) of 2.44. Although the uptake was not strongly suggestive of an aggressive malignancy, a low-grade neoplasm could not be excluded. Video-assisted thoracoscopic wedge resection was therefore performed. Histological examination revealed bland spindle-cell proliferation arranged in fascicles, with mitotic activity of < 1 per 10 high-power fields and a Ki-67 labeling index of approximately 2%. The spindle cells expressed desmin, vimentin, and estrogen receptor, whereas focal TTF-1 staining was restricted to entrapped bronchial epithelial cells. In conjunction with the bilateral multifocal pulmonary distribution and remote history of uterine leiomyoma, these findings supported a clinicopathological diagnosis of pulmonary BML. The patient recovered uneventfully without endocrine therapy. During postoperative telephone follow-up, she reported no new discomfort, and regular chest CT surveillance was recommended.

**Conclusion:**

Pulmonary BML should be considered in the differential diagnosis of multiple pulmonary nodules in women with a remote history of uterine leiomyoma. Mild FDG uptake cannot distinguish pulmonary BML from a low-grade malignancy. Integrated clinical, radiological, and pathological assessment is therefore required to establish the diagnosis and avoid unnecessary treatment.

## Introduction

Benign metastasizing leiomyoma is a rare condition characterized by histologically bland smooth-muscle lesions at extrauterine sites in patients with a history of uterine leiomyoma, with the lungs being the most commonly involved organ (3, 4, 11).

Although benign metastasizing leiomyoma (BML) remains rare, a recent systematic review included 213 case studies involving 277 patients (5). Pulmonary lesions may be detected decades after uterine surgery, and intervals of approximately 30 and 33 years have previously been reported (12, 13). Therefore, a 30-year interval should be regarded as an uncommon long-latency presentation rather than an unprecedented event.

The degree of ^18^F-FDG uptake in pulmonary BML is variable and cannot independently distinguish BML from malignancy (5, 6). We report a clinicopathologically diagnosed case of pulmonary BML detected 30 years after hysterectomy, presenting with bilateral nodular-cystic pulmonary lesions and mild ^18^F-FDG uptake with a maximum standardized uptake value (SUVmax) of 2.44.

## Case presentation

### Patient information

A 59-year-old postmenopausal Chinese woman (G2P1) was admitted to our hospital because of worsening exertional chest tightness and dyspnea during the preceding 3 months. Her medical history included recurrent mild chest tightness and shortness of breath for approximately 20 years, which had progressively worsened recently. As the symptoms were mild and intermittent, she did not previously seek medical attention or undergo thoracic imaging. Her past medical history included grade 2 hypertension for 10 years, well-controlled by oral nifedipine and losartan. She underwent total hysterectomy for uterine leiomyoma 30 years prior to admission, with no history of fibroid morcellation, thoracic surgery, dust exposure, smoking, or alcohol consumption. No family history of malignant tumors was recorded.

### Clinical manifestations

The patient reported exertional chest tightness and shortness of breath for two decades, without cough, hemoptysis, fever, night sweats, or weight loss. Her symptoms had worsened during mild physical activity over the preceding 3 months and were relieved by rest. Physical examination was unremarkable. Breath sounds were clear bilaterally, without rales or rhonchi. Cardiac and abdominal examinations were unremarkable, and no superficial lymphadenopathy was detected.

### Diagnostic assessment

No chest radiograph was obtained during the current diagnostic episode. Noncontrast chest CT was selected as the initial thoracic imaging examination because detailed characterization of the distribution and morphology of the bilateral pulmonary lesions was required.

**Figure 1:**
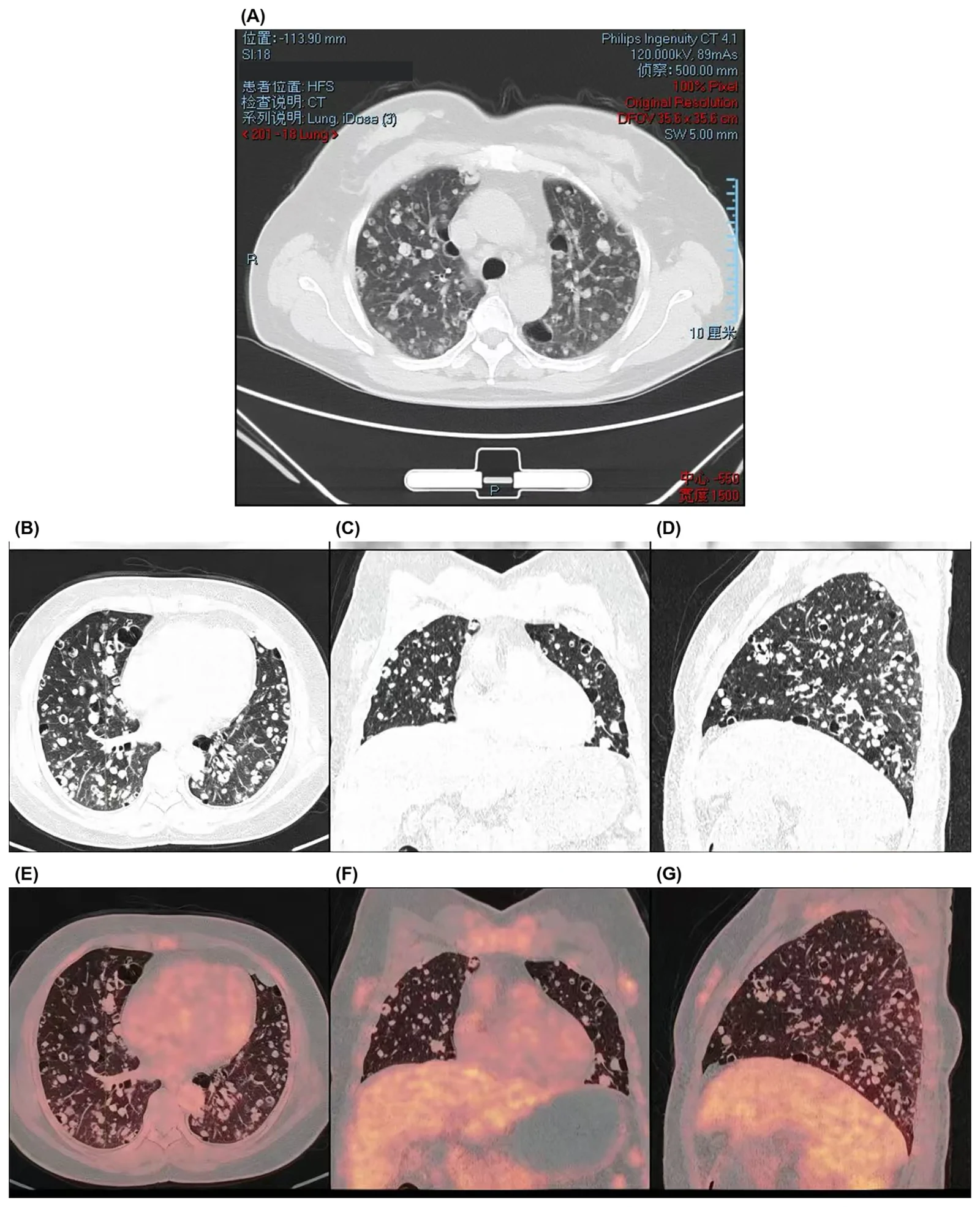
Chest CT and ^18^F-FDG PET/CT findings. **(A)** Representative axial noncontrast chest CT image shows diffuse bilateral pulmonary nodules admixed with thin-walled cystic or cavitary lesions. **(B–D)** Axial, coronal, and sagittal CT components of the PET/CT examination show the bilateral nodular-cystic distribution. **(E–G)** Corresponding fused ^18^F-FDG PET/CT images demonstrate mild heterogeneous FDG uptake in the dominant pulmonary lesion (SUVmax 2.44); mild nodal and diffuse bone-marrow uptake were interpreted as likely reactive changes.

On 7 May 2026, noncontrast chest CT showed diffuse bilateral pulmonary nodules of varying sizes admixed with thin-walled cystic or cavitary lesions, bilateral pleural thickening, and multiple subcentimeter mediastinal lymph nodes (**Figure 1A**). Based on this imaging pattern, the initial differential diagnoses included metastatic malignancy, pulmonary fungal infection, tuberculosis, and benign pulmonary proliferative disorders.

Following the CT examination, serum tumor markers, including AFP, CEA, CA-series markers, NSE, and SCC-Ag, were within the reference ranges. Serum 1,3-β-d-glucan and T-SPOT.TB tests were negative. These findings did not exclude malignancy or infection.

On 11 May 2026, ^18^F-FDG PET/CT demonstrated mild, heterogeneous FDG uptake in the bilateral pulmonary lesions, with an SUVmax of 2.44 in the dominant lesion. No focal extrapulmonary hypermetabolic lesion suspicious for a primary malignancy was identified. Mild uptake in several small cervical, mediastinal, and hilar lymph nodes and diffuse mild bone-marrow uptake were interpreted radiologically as likely reactive changes. Quantitative hepatic background SUV was unavailable in the archived report (**Figures 1B–G**).

On 13 May 2026, bronchoscopy and endobronchial ultrasound were performed to obtain tissue and microbiological samples through a less invasive approach. The bronchial mucosa appeared normal, without an endobronchial mass. Transbronchial lung biopsy and mediastinal lymph-node sampling showed chronic inflammatory infiltration, carbon deposition, and reactive lymph-node hyperplasia, without definite evidence of malignancy or fungal infection. Aspergillus galactomannan antigen and acid-fast bacillus testing of bronchoalveolar lavage fluid (BALF) were negative, and BALF next-generation sequencing did not identify a clinically significant pathogen. These results reduced the likelihood of active infection but did not establish a definitive diagnosis.

As the bronchoscopic examinations were nondiagnostic and malignancy could not be excluded, video-assisted thoracoscopic wedge resection was performed on 20 May 2026 to obtain adequate tissue. The integrated morphological, immunohistochemical, clinical, and radiological findings supported a clinicopathological diagnosis of pulmonary benign metastasizing leiomyoma.

### Multidisciplinary discussion and surgical intervention

After nondiagnostic bronchoscopic sampling, a multidisciplinary discussion recommended video-assisted thoracoscopic wedge resection to obtain adequate tissue for diagnosis. On 20 May 2026, the patient underwent right middle-lobe wedge resection and pleural adhesiolysis in the left lateral decubitus position through a 5-cm axillary incision. Estimated blood loss was approximately 10 mL, and no blood transfusion was required. Four well-circumscribed gray-white nodules were sampled. Intraoperative frozen-section examination showed no definite evidence of malignancy.

### Histopathology and immunohistochemistry

**Figure 2:**
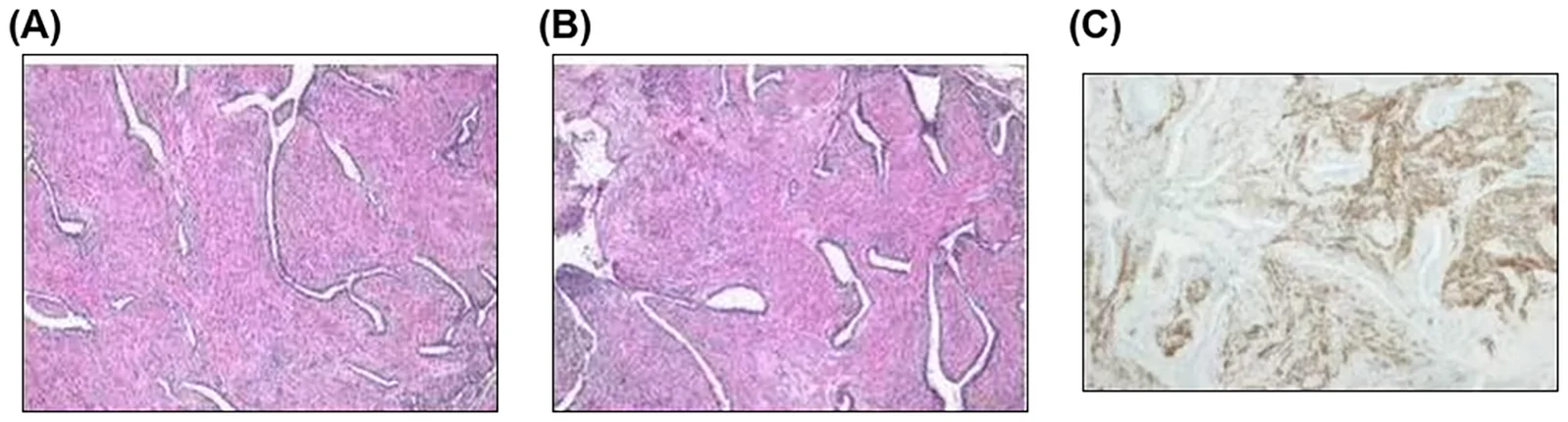
Archived pulmonary pathological images. **(A, B)** Representative hematoxylin-and-eosin-stained microscopic fields show bland spindle-cell proliferation arranged in fascicles. **(C)** Archived desmin immunohistochemistry shows positive cytoplasmic staining in the spindle-cell population. The panels were extracted from archived pathology reports. Original high-resolution photomicrographs, magnification information, and scale bars were unavailable.

Gross examination of the wedge-resection specimen revealed four well-circumscribed gray-white nodules measuring 0.5–1.2 cm in maximum diameter. Microscopically, the lesions were composed of bland spindle cells arranged in intersecting fascicles, without significant cytological atypia or tumor cell necrosis. Mitotic activity was low (< 1 mitosis per 10 high-power fields) (**Figures 2A, B**).

Immunohistochemically, the spindle cells showed diffuse cytoplasmic expression of vimentin and desmin; representative desmin staining is shown in **Figure 2C**. The spindle cells showed strong nuclear expression of estrogen receptor. The Ki-67 labeling index was approximately 2%. Focal TTF-1 staining was restricted to entrapped bronchial epithelial cells rather than the spindle-cell population.

In view of the benign smooth-muscle morphology, hormone-receptor expression, bilateral multifocal pulmonary distribution, and remote history of uterine leiomyoma, the integrated clinicopathological findings supported a diagnosis of pulmonary benign metastasizing leiomyoma. However, the original uterine specimen was unavailable for direct histological comparison, and molecular clonality analysis was not performed; therefore, a shared clonal origin of the uterine and pulmonary lesions could not be directly demonstrated.

### Postoperative course and follow-up

The patient recovered uneventfully and was discharged on 26 May 2026. No postoperative complications occurred, and no adjuvant endocrine therapy was administered. During postoperative telephone follow-up, the patient reported no new discomfort or clinical deterioration. No postoperative chest CT was available; therefore, the radiological stability of the residual bilateral pulmonary nodules could not be assessed. Chest CT surveillance every 6 months was recommended. The chronology of the patient's clinical course is summarized in **Table 1**.

**Table 1:** Timeline of clinical events.

Time	Clinical event
30 years before admission	The patient underwent total hysterectomy for uterine leiomyoma. No history of uterine fibroid morcellation was reported.
Approximately 20 years before admission	The patient began to experience intermittent exertional chest tightness and dyspnea. The symptoms were mild and relieved after rest, and no specific medical evaluation was performed at that time.
3 months before admission	Chest tightness and dyspnea worsened, especially during mild physical activity, prompting further medical evaluation.
7 May 2026	Chest noncontrast CT revealed diffuse bilateral pulmonary nodules mixed with thin-walled cystic lesions, bilateral pleural thickening, and small mediastinal lymph nodes.
11 May 2026	^18^F-FDG PET/CT demonstrated mild heterogeneous FDG uptake in the dominant pulmonary lesion (SUVmax 2.44).
13 May 2026	Bronchoscopy and EBUS-guided biopsy were performed. Pathological examination revealed chronic inflammatory changes without evidence of malignancy.
20 May 2026	Video-assisted thoracoscopic surgery (VATS) wedge resection was performed for definitive diagnosis. Histopathological and immunohistochemical findings supported a clinicopathological diagnosis of pulmonary benign metastasizing leiomyoma.
26 May 2026	The patient was discharged after an uneventful postoperative recovery.
Postoperative follow-up	Telephone follow-up indicated that the patient was in good general condition without significant postoperative complications. Regular chest CT surveillance every 6 months was recommended.

### Patient perspective

During telephone follow-up, the patient stated that she was satisfied with her postoperative recovery and had experienced no new discomfort. She understood the need for continued imaging surveillance of the residual pulmonary nodules and agreed to regular follow-up.

## Discussion

### Long-latency presentation in the context of previously reported cases

Pulmonary BML occurs in women with a prior history of uterine leiomyoma, and pulmonary lesions are commonly detected several years after hysterectomy or myomectomy (7). Much longer intervals have nevertheless been reported. In the present patient, diffuse bilateral pulmonary lesions were detected 30 years after total hysterectomy. Similar intervals of approximately 30 and 33 years have been described previously (12, 13), supporting classification of this case as an uncommon, but not unprecedented, long-latency presentation.

This patient had recurrent exertional chest tightness and dyspnea for 20 years, with progressive exacerbation over 3 months. A series of auxiliary tests were initially performed to exclude common differential diseases. Tumor marker panel (AFP, CEA, CA series, NSE, SCC-Ag) was entirely within normal limits; serum 1,3-β-d-glucan and BALF Aspergillus GM antigen were negative. The negative fungal and tuberculosis-related examinations reduced the likelihood of active pulmonary infection but did not independently exclude these diagnoses. These negative laboratory findings narrowed the diagnostic scope, while the diffuse nodular-cystic lesions on chest CT still could not be distinguished from metastatic malignant tumors. Clinical severity is variable; although many patients are asymptomatic or mildly symptomatic, rare cases may progress to severe hypoxemic respiratory failure (10).

### Mild ^18^F-FDG uptake (SUVmax = 2.44): an imaging diagnostic pitfall

Preoperative chest noncontrast CT revealed diffuse bilateral uneven soft-tissue nodules mixed with thin-walled cystic cavities, bilateral pleural thickening, and subcentimeter mediastinal lymph nodes, leading radiologists to first suspect metastatic tumor or fungal infection. Further whole-body ^18^F-FDG PET/CT demonstrated mild heterogeneous hypermetabolism in the dominant pulmonary nodule, with an SUVmax of 2.44. Pulmonary BML can mimic metastatic disease, including in patients with a concurrent gynecologic malignancy, and tissue biopsy is crucial when imaging and pathological findings are discordant (2).

Published long-latency cases do not support a universal SUVmax cutoff below 2.0. As summarized in **Table 2**, PET/CT was not reported in most of the identified reports; one congress abstract described abnormal FDG uptake without providing an SUV value, whereas another 30-year case showed no FDG uptake. These limited and discordant observations do not establish a characteristic metabolic pattern for long-latency pulmonary BML. Accordingly, the SUVmax of 2.44 in our patient should be described as mild uptake rather than as uniquely or unusually elevated uptake. PET/CT did not identify a focal extrapulmonary hypermetabolic lesion suspicious for a primary malignancy; mild cervical, mediastinal, and hilar nodal uptake and diffuse mild bone-marrow uptake were interpreted radiologically as likely reactive changes.

**Table 2:** Reported pulmonary benign metastasizing leiomyoma cases in which pulmonary lesions were detected at least 25 years after uterine surgery.

Reference (age)	Uterine surgery/interval^a^	Thoracic and other imaging	PET/CT	Diagnostic evidence	Management	Reported outcome
Avedissian et al.^b^ (14) (61 years)	Hysterectomy with unilateral oophorectomy; 33 years before pathological diagnosis. Abnormal chest radiograph about 4 years earlier	Diffuse subcentimeter pulmonary nodules in a random distribution	NR	Transbronchial biopsy: bland spindle cells without mitoses or necrosis; SMA-positive	Observation; no treatment planned	Occasional nonproductive cough initially; asymptomatic with normal spirometry at diagnostic reevaluation; later outcome NR
Huang et al. (15) (44 years)	Resection of uterine leiomyoma; 26 years	Incidental solitary LLL nodule (2.5 cm × 1.5 cm × 1.5 cm) found during evaluation of a biopsy-proven astrocytoma	NR	Wedge specimen: bland spindle cells without mitotic activity; SMA-, ER-, and vimentin-positive	Pulmonary wedge resection	NR
Jeon et al. (12) (56 years)	Total hysterectomy; 30 years	Two cystic LUL lesions (largest 5.5 cm × 4.5 cm) and a tiny LLL nodule; initially considered congenital cystic adenomatoid malformation	NR	VATS wedge specimens: smooth-muscle proliferation without mitoses, pleomorphism, or necrosis; entrapped bronchiolar structures	VATS wedge resection after 1 y of radiologic stability.	No recurrence during 1-y follow-up.
Raś et al. (16) (53 years)	Myomectomy; 26 years	Multiple pulmonary nodules; concomitant uterine, parametrial, and periappendiceal lesions	NR	Pulmonary wedge specimens: bland smooth-muscle nodules; no atypia or necrosis; ≤ 1 mitosis/10 HPF; desmin- and ER-positive; Ki-67 < 1%	Gynecologic surgery followed by pulmonary wedge resection through thoracotomy.	Lungs re-expanded; discharged on postoperative day 3; long-term follow-up NR.
Lopes et al.^b^ (17) (66 years)	Hysterectomy for benign leiomyoma; 26 years	Routine chest radiograph showed a right nodule; CT showed a homogeneous solitary top-lobe nodule	Abnormal FDG uptake; no other significant abnormal uptake; SUV NR	top-lobe resection reported as pulmonary BML; detailed morphology/IHC NR in the abstract	top-lobe resection	No lesion on CT at 6 months; asymptomatic with normal chest radiograph at 1 year
Costa et al. (13) (59 years)	Hysterectomy at age 26; 33 years	Multiple bilateral well-demarcated solid nodules; largest 1.2 cm × 0.9 cm; bilateral apical pleural thickening; no enlarged mediastinal nodes	NR	Segmentectomy: spindle-cell fascicles with entrapped respiratory epithelium; no atypia, mitoses, or necrosis; actin-, desmin-, caldesmon-, ER-, and PR-positive; Ki-67 < 5%	Segmentectomy of the largest left-lung nodule	Pulmonary symptoms regressed; follow-up CT planned at 6 months
Naia et al. (18) (72 years)	Total hysterectomy; 30 years	At least 50 well-defined 1–10-mm nodules involving all lobes, with mosaic attenuation	No FDG uptake	VATS wedge specimens: well-differentiated smooth-muscle tumors with little/no atypia and almost no mitoses; actin-, desmin-, caldesmon-, ER-, and PR-positive	Right-lung VATS wedge resection; letrozole initiated	Nodules stable on CT at 8 months before surgery; letrozole well tolerated; later outcome NR
Romero Ríos et al.^c^ (1) (74 years)	Hysterectomy at age 43; article reports approximately 33 years, whereas stated ages imply about 31 years	Multiple bilateral well-defined solid nodules (5–9 mm); no mediastinal/hilar lymphadenopathy; recurrent pelvic mass and possible iliocaval extension	NR	No pulmonary biopsy. Prior pelvic mass: cellular leiomyoma with bland spindle cells, low mitotic activity, and SMA- and desmin-positive	Multidisciplinary active surveillance	Asymptomatic (ECOG 0); follow-up planned at 3 months
Present case (59 years)	Total hysterectomy; 30 years; no reported morcellation	Diffuse bilateral nodules admixed with thin-walled cystic/cavitary lesions; pleural thickening and small mediastinal nodes	Mild heterogeneous uptake; dominant-lesion SUVmax 2.44; mild nodal and marrow uptake interpreted as likely reactive	VATS wedge specimens: bland spindle-cell fascicles; no significant atypia or tumor-cell necrosis; < 1 mitosis/10 HPF; vimentin, desmin, and ER positive; Ki-67 about 2%; TTF-1 restricted to entrapped epithelium	Right-top-lobe VATS wedge resection; no adjuvant endocrine therapy	No new symptoms on telephone follow-up; no postoperative CT available

BML, benign metastasizing leiomyoma; ECOG, Eastern Cooperative Oncology Group; ER, estrogen receptor; HPF, high-power field; LLL, left lower lobe; LUL, left upper lobe; NR, not reported; PET/CT, positron-emission tomography/computed tomography; PR, progesterone receptor; SMA, smooth muscle actin; SUVmax, maximum standardized uptake value; VATS, video-assisted thoracoscopic surgery.

a Interval denotes the time from uterine surgery to first reported detection/presentation of pulmonary lesions, unless otherwise specified.

b Published only as a meeting/congress abstract.

c Pulmonary BML was clinically/radiologically suspected without lung biopsy and should not be treated as pathologically confirmed. Goto et al. (19) was excluded because the lung nodules appeared around the time of thyroid surgery at age 40—approximately 4 years after hysterectomy—and were merely recognized histologically about 27 years later.

### Bronchoscopic biopsy limitations and necessity of surgical resection

Minimally invasive EBUS-guided transbronchial lung and mediastinal lymph node biopsy was performed for preliminary diagnosis, but only chronic inflammatory infiltration, carbon deposition, and reactive lymph node hyperplasia were observed, without evidence of tumor. BALF NGS only detected low-abundance commensal bacteria, with no pathogenic fungi or malignant genetic alterations identified.

The nondiagnostic result of bronchoscopic biopsy can be explained by the focal and isolated distribution of BML nodules within the lung parenchyma, which can make small lesions difficult to sample through a transbronchial approach (8, 9).

### Pathological diagnostic traps: bland spindle cells and focal TTF-1 expression

Gross specimens from VATS resection showed multiple well-circumscribed gray-white nodules with clear boundaries. Microscopically, the lesions consisted of fascicles of bland spindle smooth muscle cells without nuclear atypia, necrosis, or active mitosis (mitoses < 1/10 HPF). Immunohistochemistry showed diffuse vimentin and desmin positivity supporting smooth-muscle differentiation, strong nuclear ER expression consistent with the hormone-dependent characteristics of leiomyoma, and a low Ki-67 proliferation index of approximately 2%, providing additional evidence of low proliferative activity (8, 9).

Focal TTF-1 staining was confined to entrapped bronchial epithelial cells rather than the spindle-cell population. Careful assessment of the cellular localization of staining was therefore important to avoid misinterpretation. We confirmed that TTF-1 reactivity originated from entrapped normal bronchial epithelial cells within smooth muscle proliferative lesions, rather than tumor cell expression. Combined with the patient’s long-term uterine leiomyoma medical history, the department reviewed all slices and consulted pathologists at a higher-level hospital, leading to revision of the final diagnosis to multiple pulmonary benign metastasizing leiomyoma. This pathological pitfall should be emphasized in clinical and pathological practice.

The principal pathological differential diagnoses included metastatic leiomyosarcoma, primary pulmonary leiomyoma, and pulmonary lymphangioleiomyomatosis. Metastatic leiomyosarcoma was considered less likely because the pulmonary lesions showed bland cytology, very low mitotic activity, no tumor cell necrosis, and a low Ki-67 proliferation index. Nevertheless, an occult uterine smooth-muscle tumor of uncertain malignant potential or a well-differentiated leiomyosarcoma could not be excluded with absolute certainty because the original uterine specimen was unavailable for review.

Primary pulmonary leiomyoma can show similar smooth-muscle morphology but was considered less likely because the lesions were bilateral and multifocal and occurred in a patient with a previous uterine leiomyoma. This clinical distribution favored pulmonary BML, although uterine origin could not be proven without direct pathological or molecular comparison.

Pulmonary lymphangioleiomyomatosis was considered because thin-walled cystic lesions were present on CT. However, the resected lesions formed discrete, well-circumscribed nodules composed of fascicular spindle cells rather than the diffuse interstitial, peribronchiolar, and perivascular smooth-muscle proliferation characteristic of lymphangioleiomyomatosis. As an expanded immunohistochemical panel was unavailable, the distinction was based primarily on the observed morphology and clinicoradiological distribution.

Solitary fibrous tumor (SFT) and inflammatory myofibroblastic tumor (IMT) were also considered in the differential diagnosis of a pulmonary spindle-cell lesion. SFT typically shows a patternless arrangement of spindle-to-ovoid cells in a collagenous stroma with branching staghorn-type vessels and characteristically demonstrates nuclear STAT6 expression, often with CD34 positivity, reflecting the NAB2-STAT6 fusion (20). By contrast, the present lesions showed intersecting fascicles of bland smooth-muscle cells with diffuse desmin and vimentin expression and strong ER positivity. IMT typically comprises myofibroblastic spindle cells in a myxoid or collagenized stroma with a conspicuous inflammatory infiltrate; subsets harbor ALK, ROS1, or other kinase rearrangements (21). The absence of a prominent inflammatory background and the hormone-receptor-positive smooth-muscle phenotype favored BML over IMT. However, because STAT6, CD34, ALK, and molecular testing were unavailable, SFT and IMT could not be excluded solely by an expanded ancillary panel; the final diagnosis therefore relied on integrated morphology, bilateral multifocal distribution, and the remote history of uterine leiomyoma.

### Pathogenesis of pulmonary BML

Three mainstream pathogenetic hypotheses are widely recognized: hematogenous dissemination of smooth muscle cells during gynecologic surgery, intravascular leiomyomatosis extension to the thorax, and multifocal hormone-sensitive mesenchymal smooth muscle metaplasia (11). However, the mechanism responsible for the pulmonary lesions could not be determined because the original uterine specimen was unavailable and molecular clonality analysis was not performed. Estrogen-receptor expression supported hormone responsiveness but did not establish the mechanism responsible for the pulmonary lesions.

### Comparison with previously reported long-latency pulmonary BML cases

We identified published pulmonary BML reports in which pulmonary lesions were detected at least 25 years after uterine surgery (**Table 2**). Presentations ranged from a solitary nodule to diffuse bilateral solid or cystic disease, sometimes with extrapulmonary leiomyomatous lesions. PET/CT was usually unreported: one 26-year case showed abnormal FDG uptake without an SUV value, whereas one 30-year case showed no uptake. These discordant data do not establish a characteristic metabolic pattern. Accordingly, the SUVmax of 2.44 in our patient is best described as representing mild uptake that did not reliably distinguish BML from a low-grade malignancy. Management varied from observation to resection and hormonal therapy, and follow-up was often incomplete. A 30-year interval is therefore uncommon but not unprecedented.

### Clinical management strategy

There is no unified standardized treatment guideline for pulmonary BML. As postoperative assessment was limited to telephone follow-up and no postoperative chest CT was available, the stability of the residual bilateral pulmonary lesions could not be evaluated. The patient reported no new symptoms and did not receive adjuvant endocrine therapy. Regular chest CT surveillance every 6 months was recommended; endocrine treatment may be considered if subsequent imaging demonstrates lesion enlargement or if respiratory symptoms worsen.

### Strengths and limitations

The principal strength of this report is the detailed description of pulmonary benign metastasizing leiomyoma presenting approximately 30 years after hysterectomy, together with an integrated radiological, bronchoscopic, histopathological, and immunohistochemical assessment. The case also illustrates that mild FDG uptake in pulmonary nodules may contribute to diagnostic uncertainty and should not be interpreted independently of the clinical history and pathological findings.

Several limitations should be acknowledged. First, the original uterine surgical specimen and histopathological slides from approximately 30 years earlier were unavailable for direct comparison with the pulmonary lesions. Therefore, the uterine origin of the pulmonary smooth muscle lesions could not be confirmed by morphological comparison or molecular clonality analysis. Second, additional immunohistochemical markers, including smooth muscle actin and progesterone receptor, were not available. Nevertheless, the diagnosis was supported by the bland smooth muscle morphology, diffuse desmin and vimentin expression, strong ER positivity, low Ki-67 proliferation index, and the remote history of uterine leiomyoma. Third, quantitative hepatic background SUVmean and SUVmax were unavailable from the archived PET/CT data, preventing calculation of a lesion-to-liver uptake ratio. Fourth, postoperative assessment was limited to telephone follow-up, and no postoperative follow-up CT was available to evaluate the long-term behavior of the residual bilateral pulmonary nodules. Original high-resolution histopathological and immunohistochemical photomicrographs were unavailable. Only two low-resolution H&E images embedded in the archived pathology report could be retrieved. This limited the quality of the pathological figure and the independent visual assessment of the reported immunohistochemical findings. Finally, the literature review was narrative rather than systematic and was restricted primarily to English-language reports; therefore, relevant cases may have been omitted.

An additional diagnostic limitation was the absence of STAT6, CD34, and ALK immunohistochemistry and molecular testing for NAB2-STAT6 or kinase fusions; therefore, SFT and IMT could not be directly excluded using those ancillary studies.

## Conclusion

This case illustrates an uncommon long-latency presentation of pulmonary BML detected 30 years after hysterectomy. The dominant pulmonary lesion showed mild ^18^F-FDG uptake with an SUVmax of 2.44, which did not reliably distinguish BML from a low-grade malignancy. Bronchoscopic sampling was nondiagnostic, and the clinicopathological diagnosis was supported by surgical biopsy, bland smooth-muscle morphology, desmin and vimentin expression, estrogen-receptor positivity, and a low Ki-67 labeling index. Pulmonary BML should remain in the differential diagnosis of bilateral pulmonary nodules in women with a remote history of uterine leiomyoma. As the original uterine specimen and molecular clonality evidence were unavailable, the uterine origin of the pulmonary lesions could not be directly demonstrated. Continued imaging surveillance is required to assess the behavior of the residual pulmonary nodules.

## Data Availability

The original contributions presented in the study are included in the article/supplementary material. Further inquiries can be directed to the corresponding author.

## References

[1] Ríos CKR Alemán BRL Zamora AS . Late-onset pulmonary benign metastasizing leiomyoma 3 decades after hysterectomy: A case report. Radiol Case Rep. (2026) 21:1360–4. doi: 10.1016/j.radcr.2025.12.008 41551098 PMC12809086

[2] Yoshida T Nagao T Ozawa R Hida K . Benign metastasizing leiomyoma with endometrial carcinoma, with a differential diagnosis of metastatic lung cancer. BMJ Case Rep. (2021) 14:e240922. doi: 10.1136/bcr-2020-240922 33846186 PMC8048007

[3] Perri T Komem DA Apter S Inbar Y Dick-Necula D Levin G . Spontaneous versus morcellator-related benign metastasizing leiomyoma-A retrospective study. Int J Gynaecol Obstet. (2022) 157:110–4. doi: 10.1002/ijgo.13830 34270803

[4] André D Gouveia F Luís H Caldeira M Perneta F Gonçalves MJ . Benign metastasizing leiomyoma - a case of benign metastasis. JRSM Open. (2021) 12:20542704211064482. doi: 10.1177/20542704211064482 34925863 PMC8674719

[5] Karoń K Szczęsny N Piątkowski K Bornio E Grabowski W Plata H . Benign metastasizing leiomyoma: a systematic review of diagnosis, management, and outcomes. Chin Clin Oncol. (2026) 15:37. doi: 10.21037/cco-25-40 42145021

[6] Gosavi A Shah S Purandare NC Puranik A Agrawal A Rangarajan V . Benign metastasizing leiomyoma and findings on fluorodeoxyglucose positron emission tomography/contrast-enhanced computed tomography. Indian J Nucl Med. (2022) 37:68–70. doi: 10.4103/ijnm.ijnm_118_21 35478683 PMC9037878

[7] Tirmazy S Farooq Latif M Nadir Salih G Ahmed B Talib Erabia W . A rare case of pulmonary benign metastasizing leiomyomatosis in a woman with a previous history of hysterectomy for uterine fibroids. Clin Med (Lond). (2023) 23:78–80. doi: 10.7861/clinmed.2022-0468 36697018 PMC11046539

[8] Su H Fan R Yang H You Y Zhu L Feng F . Pulmonary benign metastasizing leiomyoma in patients aged 45 years and younger: clinical features and novelty in treatment. BMC Pulm Med. (2023) 23:168. doi: 10.1186/s12890-023-02406-7 37189093 PMC10186780

[9] Fan R Feng F Yang H Xu K Li S You Y . Pulmonary benign metastasizing leiomyomas: a case series of 23 patients at a single facility. BMC Pulm Med. (2020) 20:292. doi: 10.1186/s12890-020-01330-4 33172427 PMC7653756

[10] Araujo NM Cardoso IMDS Jatobá TKADS Mencato Sabey LP Teixeira AKAAF Tojal AST . Rare manifestation of pulmonary benign metastasizing leiomyoma: Respiratory failure. Respir Med Case Rep. (2024) 50:102053. doi: 10.1016/j.rmcr.2024.102053 38881776 PMC11176773

[11] Awonuga AO Shavell VI Imudia AN Rotas M Diamond MP Puscheck EE . Pathogenesis of benign metastasizing leiomyoma: a review. Obstet Gynecol Surv. (2010) 65:189–95. doi: 10.1097/OGX.0b013e3181d60f93 20214834

[12] Jeon HW Choi SH Sung SW Park JK . Pulmonary benign metastasizing leiomyoma: report of three cases. World J Surg Oncol. (2013) 11:281. doi: 10.1186/1477-7819-11-281 24139514 PMC3828480

[13] Costa R Santos L Neves JBV Reis C . Pulmonary benign metastasizing leiomyoma: case report of late occurrence in a postmenopausal patient. Rev Bras Cancerol. (2022) 68:e-092408. doi: 10.32635/2176-9745.RBC.2022v68n1.2408

[14] Avedissian LS Hnatiuk OW Frenia DS . Benign metastasizing leiomyoma-an unusual cause of diffuse pulmonary nodules. Chest. (2004) 126:989S. doi: 10.1378/chest.126.4_MeetingAbstracts.989S-a19292919

[15] Huang H Balos L Chen F . Pulmonary benign metastasizing leiomyoma appears 26 years after resection of uterine leiomyoma. N A J Med Sci. (2012) 5:55–7. doi: 10.7156/v5i1p055

[16] Raś R Książek M Barnaś E Skręt-Magierło J Kąziołka W Fudali L . Benign metastasizing leiomyoma in triple location: lungs, parametria and appendix. Prz Menopauzalny. (2016) 15:117–21. doi: 10.5114/pm.2016.61195 27582687 PMC4993987

[17] Lopes K Barbosa M Garcez Marques H Camacho ME . Pulmonary benign metastasizing leiomyoma in a postmenopausal woman [abstract PE 004. Pulmonology. (2019) 25:123.

[18] Naia JV Pimenta D Paiva A Costa R Souto de Moura C Pereira R . When benign leiomyomas metastasize to the lungs-a case report. Monaldi Arch Chest Dis. (2023) 93:2488. doi: 10.4081/monaldi.2023.2488 36786165

[19] Goto M Ichikawa Y Tsubouchi H Fukumoto K Mori S . A case of pulmonary benign metastasizing leiomyoma presented with pulmonary metastases of thyroid cancer. Nihon Rinsho Geka Gakkai Zasshi. (2024) 85:1012–7. doi: 10.3919/jjsa.85.1012

[20] Doyle LA Vivero M Fletcher CD Mertens F Hornick JL . Nuclear expression of STAT6 distinguishes solitary fibrous tumor from histologic mimics. Mod Pathol. (2014) 27:390–5. doi: 10.1038/modpathol.2013.164 24030747

[21] Antonescu CR Suurmeijer AJH Zhang L Sung YS Jungbluth AA Travis WD . Molecular characterization of inflammatory myofibroblastic tumors with frequent ALK and ROS1 gene fusions and rare novel RET rearrangement. Am J Surg Pathol. (2015) 39:957–67. doi: 10.1097/PAS.0000000000000404 25723109 PMC4465992

